# Tradeoffs in climate adaptation? Early data suggest reducing livelihood risks does not change health risks for mobile herders

**DOI:** 10.1016/j.joclim.2026.100707

**Published:** 2026-07-10

**Authors:** Anne C. Pisor, Deepti Singh, Dalmas Ochieng’ Omia, Dismas Oketch, Isaac Ngere, Eric Osoro, M. Kariuki Njenga

**Affiliations:** a Department of Anthropology and Social Science Research Institute, Penn State University, 237J Welch Building, 137 Fischer Rd, University Park, PA 16803, USA; b Department of Human Behavior, Ecology, and Culture, Max Planck Institute for Evolutionary Anthropology, Deutscher Platz 6, Leipzig 04103, Germany; c School of the Environment, VSCI 230D, 14204 NE Salmon Creek Ave, Washington State University, Vancouver, WA 98686, USA; d Department of Anthropology, Gender and African Studies, University of Nairobi, P. O. Box 30197-00100, Museum Hill, Nairobi, Kenya; e Paul G. Allen School for Global Health, Washington State University, P.O. Box 647010, Pullman, WA 99164-7010, USA

**Keywords:** Climate adaptation, Mobility, Drought, Noncommunicable disease, Pastoralists, East Africa

## Abstract

**Introduction:**

Climate events like drought adversely affect the health and livelihoods of mobile or migrant communities. Individuals take various actions to reduce these risks – but how does climate adaptation in one domain, like livelihood, potentially increase risk in another, like health? How might actions to reduce health risks now, like by improving food security, increase health risks later, like to mental health?

**Methods:**

We present findings from individual surveys (n = 90) and in-depth interviews (n = 16) with Rendille people in northern Kenya that were conducted to understand the health impacts of and responses to hydroclimate extremes. Like many other mobile peoples, Rendille are on the climate frontlines.

**Results:**

During a recent unprecedented drought (2020–2023), Rendille employed a range of strategies to reduce livelihood impacts and improve food security, including selling and slaughtering animals, sharing food, and going into debt to buy food. Importantly, herders changed their movement patterns, trekking an average of 7 days farther than usual with their animals in search of pasture. Some herders who moved their animals reported that being so far away increased loneliness and stress; however, we found no clear differences in self-reported health effects between herders who stayed close to home vs herders who left.

**Conclusion:**

Examining potential co-harms or tradeoffs between different adaptations – like reducing risks to livelihood at a cost to health – will help policymakers and stakeholders better evaluate actions to reduce climate-related risks, especially for mobile populations.

## Graphical abstract

**Figure:**
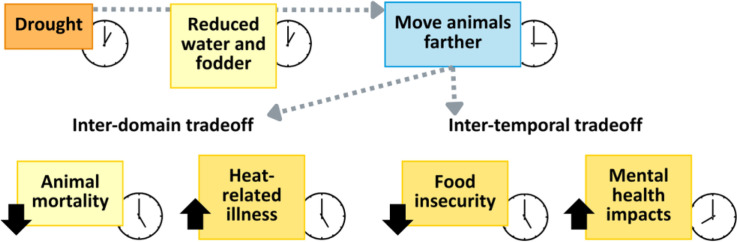
A diagram capturing the relationship, and timing, of the effects of different variables on candidate adaptations and how their effects may trade off across domains (e.g., livelihood vs health) and across time (e.g., health now vs health later).

## 1 Introduction

Climate change is altering movement and settlement patterns for both mobile and sedentary populations [1], with millions of people annually using mobility to respond to climate impacts [2]. Such movement can have myriad health impacts, from increasing food insecurity and incidence of cardiovascular disease, to negative effects on mental health [3].

Even for already-mobile populations like mobile pastoralists, droughts and floods change movement, affecting both short-term and cumulative risk factors for communicable and noncommunicable diseases [3]. High temperatures, drought, heavy rainfall, and flooding affect pasture and water access [4], leading herders to change movement patterns or diversify their livelihoods. For Rendille people, mobile pastoralists in northern Kenya, this includes adopting crop cultivation or engaging in wage labor.

**Fig. 1:**
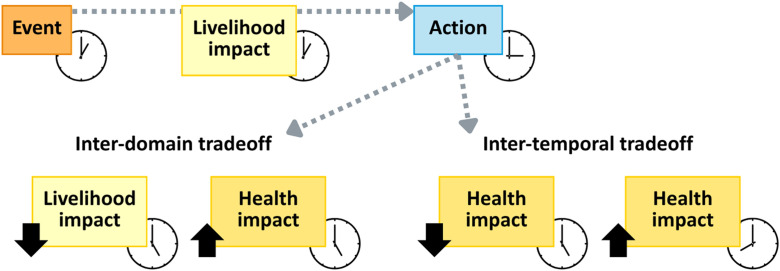
A simplified diagram of the relationship between climate events, impacts, and action. When an action reduces impact, we speak of resilience or something being adaptive. However, reducing risk to livelihood can increase adverse health impacts in the near-term (inter-domain tradeoff), or reducing risk to livelihood can reduce adverse health impacts now but increase them later (inter-temporal tradeoff).

While mobility and diversified livelihoods potentially reduce short-term negative health impacts by e.g. minimizing short-term food insecurity risks [4,5], they can increase other risks to human health in the long term (an inter-temporal tradeoff; Fig. 1). Further, mobility can reduce financial risk from any one source [6,7] but disrupt social networks and social support, impacting mental health [8] (an inter-domain tradeoff; Fig. 1). In short, climate adaptations can generate co-harms [9]; what may be adaptive in one domain can be maladaptive in another [3].

The experience of Rendille people illuminates these tradeoffs. In 2020–23, Rendille experienced the most severe drought in the last 40 years, which led to 13.2 million livestock deaths across the Horn of Africa [10]. Our interviews with Rendille reveal that drought led to experience of amplified heat-related illness and mental health impacts; while some herders changed their movement patterns, we found no clear indication this changed the health impacts they experienced. We detail those findings here, then close by outlining next steps for examining any inter-domain and inter-temporal tradeoffs in resilience and adaptation for mobile peoples.

## 2 Methods

### 2.1 Population

About 64,000 Rendille people live in northern Kenya. Most practice pastoralism, moving camels, cattle, sheep, goats, and donkeys to “fora” (grazing areas with water) at varying distances from their semi-permanent settlements (“manyatta”). Animals hold substantial value to Rendille people – they are a symbol of wealth and a source of nutrition and income. Women may herd sheep and goats close to the manyatta but generally remain within a few kilometers of settlements. Men take animals to fora near and far.

Drought is one of three “enemies” Rendille confront, alongside animal disease and raiding [11]. Climate change increased the extent and severity of the 2022 portion of the drought in East Africa [12] and additional warming could further increase the frequency, duration, and/or severity of droughts in parts of the region [13]. In recent decades, Rendille practices for responding to drought have included livestock borrowing [11,14], changing herd size or composition, moving animals to distant fora, and diversifying livelihoods via e.g., agriculture [15] or wage labor.

### 2.2 Data collection

A coauthor (DOO) and two research assistants (RAs) collected pilot data in October-November 2024. Interviews were conducted in Rendille, with translations to and from Rendille provided by local RAs. We report on (1) 16 semi- and unstructured interviews with elders and (2) surveys about the 2020–23 drought with 45 participants who stayed near the village with their animals (“stay”; n = 35 women; ages 19–74) and 45 who trekked to distant fora with their animals (“go”; n = 10 women; ages 21–79). Data collection protocols were approved by the Kenya Medical Research Institute (SERU #4405) with reliance from the Washington State University Institutional Review Board (IRB). For further details on protocol, ethics, and descriptive statistics, see Supplement.

### 2.3 Data processing and visualization

Data were processed and visualized, and descriptives calculated, using the R statistical program [16] with tidyverse [17]. We compare participants’ experiences across the “stay” and “go” groups using a combination of qualitative data, including themes and direct quotes, and quantitative data, including data visualization and descriptive statistics.

## 3 Results

**Fig. 2:**
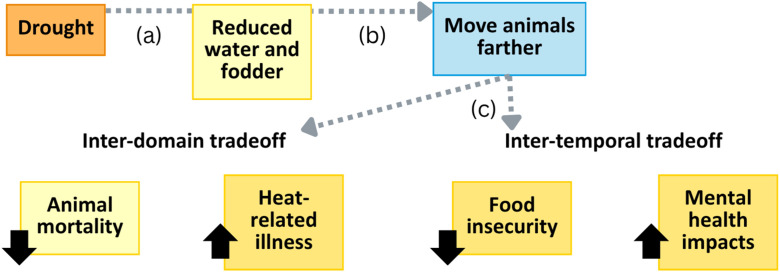
A version of Fig. 1 modified to include specific variables and relationships (a-c) discussed in this article.

Participants’ well-being – livelihoods and health – is impacted by droughts. Elders reported that droughts often kill animals: “The worst drought [is] when animals die in large numbers,” said one elder man. To minimize the impact of drought on their herds, herders “travel to distant areas in search of pasture for their animals,” said another elder man (Fig. 2a). “Despite the extreme heat, they trekked with the animals for almost 12 h a day.”

**Fig. 3:**
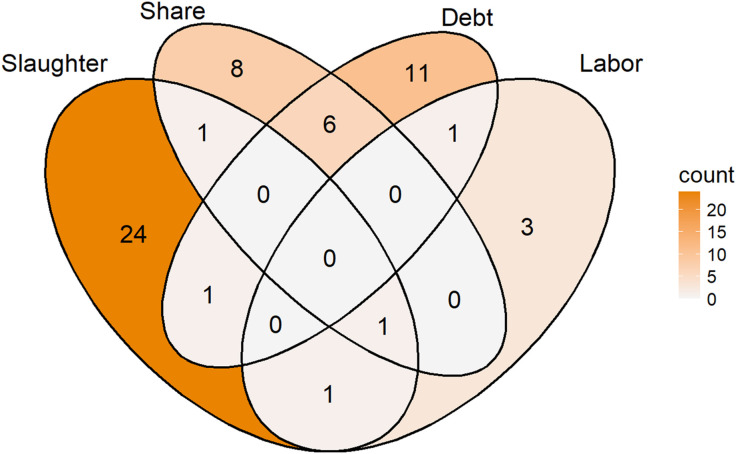
Combinations of strategies participants used when they were hungry during the 2020–23 drought. Almost all participants sold animals to buy food (n = 88; not shown). Some also used the four strategies below, described in Section 3.

Participants took action to reduce impacts to livelihoods during the 2020–23 drought. “Go” participants (n = 45) spent a median 9 months away (range 4–36) and trekked 7 days more than normal (range 2–30), up to 300 km from settlements (Fig. 2b). To protect their livelihoods and food security, 98% of participants sold livestock and two-thirds took at least one additional action, like slaughtering animals to eat, receiving gifts or loans of food or money from neighbors and family, going into debt with shops, or doing wage labor (Fig. 3).

**Fig. 4:**
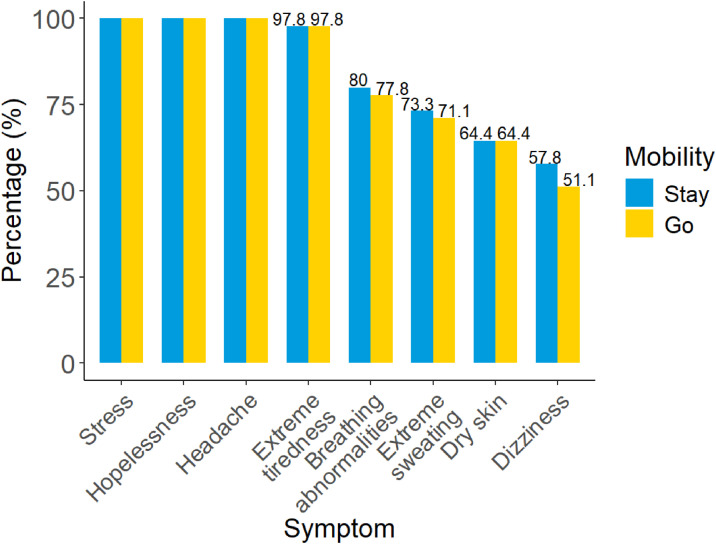
Histogram of health impacts participants reported, with “stay” participants in blue and “go” in yellow. Similar numbers of both “stay” and “go” participants recalled experiencing the symptoms listed.

Different actions did not differentially impact human health. Similar numbers of both “go” and “stay” participants reported experiencing heat-related illness symptoms at some point during the prolonged drought (Fig. 2c; Fig. 4). While slightly more “stay” participants reported dizziness, this difference was insignificant (t = −0.63, df = 88, p = 0.53). In interviews, elder women reported changed exposures to heat; one noted, “We have never experienced such hot days, and it is difficult to carry out activities outside,” and another said, “I was walking from [town] to [my village] and due to the extreme heat, I had to rest in the shade for a few hours.” Some “go” participants remarked that traveling to further, less-familiar fora increased feelings of loneliness and worry about raids (Fig. 2c), but all participants reported experiencing stress and hopelessness.

## 4 Discussion

It is well-known that climate events like drought pose a risk to health and livelihoods, and that individuals take actions to reduce these risks. What is less well understood are how, for highly-exposed populations such as mobile pastoralists, climate adaptations in one domain, like livelihood, can potentially increase risk in another, like health [3], or how actions to reduce health risks now, like improving food security, can increase health risks later, like decreased mental health [5] (Fig. 1). We know especially little about these tradeoffs in mobile or migrant peoples, who are on the climate frontlines but can be difficult to reach [18,19] and are generally underrepresented in the public health literature [18]. Understanding any tradeoffs or co-harms of responses to climate extremes is important for guiding decision-making and priority-setting for stakeholders and policymakers.

We examined potential evidence for these tradeoffs among Rendille pastoralists in northern Kenya (Fig. 2). During the 2020–23 drought, the most severe and long-lasting in 40 years, Rendille took action to reduce livelihood impacts, including trekking farther than usual with their animals in search of pasture. More than 50% of participants in both groups reported experiencing multiple negative physical and mental health outcomes, underscoring the compounding health impacts of climate extremes. However, we found no clear differences in reported experience of heat-related illness and mental health symptoms among herders who moved their animals, often hundreds of kilometers from home, vs herders who did not.

### 4.1 Limitations and future directions

There are key limitations to these preliminary observations. We rely on recall of a salient, large-scale event, but participants could experience recall bias such that e.g., brief heat-related illness symptoms are under- or overreported. We focus on herders rather than those who have fully transitioned to agriculture or wage labor, so we know less about how larger adjustments to livelihood have downstream impacts on health. Our ongoing work includes Rendille with transitioned livelihoods to better understand the health outcomes of livelihood diversification. Finally, these cross-sectional data do not have the temporal resolution to capture the detailed immediate and long-term health consequences of adaptation in different domains. We are now working with Rendille to collect longitudinal, real-time data on heat exposure; the actions Rendille people take to reduce the risk to their health and livelihoods; and downstream, cumulative consequences of these actions for their physical and mental health, to better characterize any inter-domain and inter-temporal tradeoffs of climate adaptation for mobile peoples.

## 5 Conclusion

Supporting climate adaptation or resilience means understanding any co-harms or tradeoffs in outcomes over different time scales – reducing health risks now could increase them later – and in different domains – reducing livelihood risks can increase health risks. We must especially understand any tradeoffs for mobile peoples, where choices to alter movement can have short-term and cumulative impacts on health. During the 2020–23 Horn of Africa drought, Rendille people in Kenya increased mobility to provide their animals with better pasture; however, in self-reported recall data, differences in mobility did not result in differences in mental and physical health. Our ongoing collaboration with Rendille will reveal how actions to reduce risks could affect human health in real time and cumulatively, providing a blueprint for how to better understand any benefits and costs of climate adaptation in mobile peoples, across time and across domains.

## Funders

Funding was provided by WSU Vancouver, Penn State Social Science Research Institute, and the CREID-ECA grant number U01AI151799 from National Institute of Allergy and Infectious Diseases/National Institutes of Health (NIAID/NIH).

## Declaration of competing interest

The authors declare that they have no known competing financial interests or personal relationships that could have appeared to influence the work reported in this paper.

## Appendix Supplementary materials

Image, application 1

## Footnotes

Supplementary material associated with this article can be found, in the online version, at doi:10.1016/j.joclim.2026.100707.

## References

[1] McMichael C. (2023). Climatic and environmental change, migration, and health. Annu Rev Public Health.

[2] UNHCR (United Nations High Commissioner for Refugees) (2016). Frequently asked questions on climate change and disaster displacement [Internet]. Stories.

[3] Luyten A., Winkler M.S., Ammann P., Dietler D. (2023). Health impact studies of climate change adaptation and mitigation measures – a scoping review. J Clim Change Health.

[4] Herrero M., Addison J., Bedelian C., Carabine E., Havlik P., Henderson B. (2016). Climate change and pastoralism: impacts, consequences and adaptation. Rev Sci Tech OIE.

[5] Scheelbeek P.F.D., Dangour A.D., Jarmul S., Turner G., Sietsma A.J., Minx J.C. (2021). The effects on public health of climate change adaptation responses: a systematic review of evidence from low- and middle-income countries. Environ Res Lett ERL Web Site.

[6] Hunter L.M., Luna J.K., Norton R.M. (2015). Environmental dimensions of migration. Annu Rev Sociol.

[7] Savo V., Lepofsky D., Benner J.P., Kohfeld K.E., Bailey J., Lertzman K. (2016). Observations of climate change among subsistence-oriented communities around the world. Nat Clim Change.

[8] Adger W.N., Barnett J., Brown K., Marshall N., O’Brien K. (2013). Cultural dimensions of climate change impacts and adaptation. Nat Clim Change.

[9] Scovronick N., Budolfson M., Dennig F., Errickson F., Fleurbaey M., Peng W. (2019). The impact of human health co-benefits on evaluations of global climate policy. Nat Commun.

[10] United Nations Office for the Coordination of Humanitarian Affairs (2023). Horn of Africa drought regional humanitarian overview & call to action [Internet]. Analysis.

[11] Sun X. (2017). Strengthening local safety nets as a key to enhancing the food security of pastoralists in east africa: a case study of the rendille of Northern Kenya. Afr Study Monogr Suppl Issue.

[12] Gebrechorkos S.H., Sheffield J., Vicente-Serrano S.M., Funk C., Miralles D.G., Peng J. (2025). Warming accelerates global drought severity. Nature.

[13] Gebrechorkos S.H., Taye M.T., Birhanu B., Solomon D., Demissie T. (2023). Future changes in climate and hydroclimate extremes in East Africa. Earths Future.

[14] Schlee G. (1989).

[15] Fratkin E. (2001). East african pastoralism in transition: Maasai, Boran, and Rendille cases. Afr Stud Rev.

[16] R Core Team (2025). http://www.r-project.org/.

[17] Wickham H., Averick M., Bryan J., Chang W., McGowan L.D., François R. (2019). Welcome to the tidyverse. J Open Source Softw.

[18] Jean-Richard V., Crump L., Moto Daugla D., Hattendorf J., Schelling E., Zinsstag J. (2014). The use of mobile phones for demographic surveillance of mobile pastoralists and their animals in Chad: proof of principle. Glob Health Action.

[19] Schelling E., Greter H., Kessley H., Abakar F., Naré N.B., Crump L. (2016). Human and animal health surveys among pastoralists. Rev Sci Tech OIE.

