# Supplementary material for: Tradeoffs in climate adaptation? Early data suggest reducing livelihood risks does not change health risks for mobile herders
Source: J Clim Chang Health. 2026 Jul 10;30:100707. doi: 10.1016/j.joclim.2026.100707 (PMC13380206; doi:10.1016/j.joclim.2026.100707)
Supplement: Supplementary file 1 [file mmc1.docx]

**SUPPLEMENTARY MATERIALS**

**Tradeoffs in climate adaptation? Early data suggest reducing livelihood risks does not change health risks for mobile herders**

Anne C Pisor^a,b*^, Deepti Singh^c^, Dalmas Ochieng’ Omia^d^, Dismas Oketch^e^, Isaac Ngere^e^, Eric

Osoro^e^, M. Kariuki Njenga^e^

^a^Department of Anthropology and Social Science Research Institute, Penn State University;

237J Welch Building, 137 Fischer Rd, University Park, PA 16803, USA

^b^Department of Human Behavior, Ecology, and Culture, Max Planck Institute for Evolutionary

Anthropology; Deutscher Platz 6, 04103 Leipzig, Germany

^c^School of the Environment, Washington State University, Vancouver, WA; VSCI 230D, 14204

NE Salmon Creek Ave, Vancouver, WA 98686, USA

^d^Department of Anthropology, Gender and African Studies, University of Nairobi; P. O. Box

30197-00100, Museum Hill, Nairobi, Kenya

^e^Paul G. Allen School for Global Health, Washington State University; P.O. Box

647010, Pullman, WA 99164-7010, USA

*corresponding author: pisor@psu.edu

[Appendix 1: Protocol](#_g7wyqaoy31so)

[1.1 Sampling](#_w0iex8ctjbg8)

[1.2.1 Interviews](#_96ukrflrkcx0)

[1.2.2 Survey](#_gwe2fxr8r7wx)

[1.2 Data collection](#_c8vdy7w9o9ha)

[1.2.1 Interviews](#_iox0x7fmxuvg)

[1.2.2 Survey](#_obr4romvaocz)

[1.4 Ethics](#_arssadfxu06p)

[Appendix 2: Descriptive statistics](#_vbrju3aw1ed0)

[Interview sample characteristics](#_slm3ker01poo)

[Survey sample characteristics](#_yua0ppht6us2)

[Appendix 3: Interview guide](#_lj9jo5h042zx)

[Appendix 4: Survey questions](#_7stptu5mny8y)

# Appendix 1: Protocol

For details on study population and data analytics, see main text.

## 1.1 Sampling

### 1.2.1 Interviews

Interviews were conducted with 16 elders, eight women and eight men. Our sample size of 16 was determined by saturation, as evaluated by co-author Omia. We interviewed a maximum of one interviewee per household, and efforts were made to sample across gender, villages, clans, and older ages; see below for descriptive statistics.

### 1.2.2 Survey

**Table S1.** Interviews were conducted with 90 individuals, split across four categories.

|  | **Women** | **Men** | **Total** |
| --- | --- | --- | --- |
| **Stay** | 35 | 10 | **45** |
| **Go** | 10 | 35 | **45** |
| **Total** | **45** | **45** |  |

Efforts were made to sample across villages, clans, and ages; see below for descriptive statistics.

## 1.2 Data collection

Data were collected in October and November 2024 near Laisamis, Marsabit County, Kenya.

### 1.2.1 Interviews

Co-autho Omia and two research assistants conducted semi-structured interviews in participants’ homes based on an interview guide (Appendix 3). Interviews were conducted in Rendille and were recorded, transcribed, and translated from Rendille to English. Interviews lasted approximately 1 hour and participants were paid 1,000 Kenyan shillings (approximately $7.75 in October 2024).

### 1.2.2 Survey

Two research assistants collected survey data in participants’ homes using RedCAP on portable tablets. Surveys were conducted in Rendille by RAs who were native Rendille speakers, and RAs marked participant responses in English. Interviews lasted approximately 1 hour and participants were compensated with 1,000 Kenyan shillings (approximately $7.75 in October 2024).

## 1.4 Ethics

Data collection protocols were approved by the Kenya Medical Research Institute (SERU #4405) with reliance from Washington State University IRB. Participants provided consent either with their signature or verbally, as documented by a witness signature. To promote confidentiality, survey data were attached to an anonymized participant ID number maintained by WSU; interview participants were assigned an ad-hoc ID number (e.g., elder_man_001).

# Appendix 2: Descriptive statistics

## Interview sample characteristics

Co-author Omia and two research assistants interviewed 8 female and 8 male participants, all above age 60; participant age was not consistently collected.

## Survey sample characteristics

As aforementioned, we interviewed 90 participants: 45 who stayed (30 females, 10 males) and 45 who left (10 females, 30 males). Distribution of age by gender across “stay” and “go” subsamples was as follows:

| **Subsample** | **Gender** | **Mean** | **SD** | **Min** | **Max** |
| --- | --- | --- | --- | --- | --- |
| **Stay** | **Female** | 44.5 | 12.8 | 19 | 72 |
| **Stay** | **Male** | 42.9 | 13.4 | 25 | 74 |
| **Go** | **Female** | 38.5 | 3.21 | 35 | 45 |
| **Go** | **Male** | 44.8 | 16.7 | 21 | 79 |

# Appendix 3: Interview guide

1. What are the main environment-related threats or stressors to your community over the last 20-30 years?

- Long dry periods, flash floods, and very hot days? Anything else?

What changes (frequency and intensity) have you noticed on the above threats?

2. Can you recall events that had the worst effect on the people and the livestock [how do these climate hazards affect you? who (men, women, children) are most affected?]?

- Example of notable floods and long dry periods
- Which assets and resources are most affected?

3. How did long dry periods affect your community?

- Did they affect availability of clean water for daily activities?
- Did they affect food availability?
- How were the camel, goats, and sheep affected?
- Were there impacts on the main sources of livelihood for families?
- Did these events affect cultural practices and traditions?

4. How did floods affect your community?

- Did they affect availability of clean water for daily activities?
- Did they affect food availability?
- How were the camel, goats, and sheep affected?
- Were there impacts on the main sources of livelihood for families?
- Did these events affect cultural practices and traditions?

5. How did these events affect the health of your community members?

- What were the impacts on your health and lives?
- What were the impacts on the health of morans/herders?
- How did these affect where and how far they travel for fora?
- What were the impacts on the health of women?
- How did these events affect the daily activities (e.g. water collection, etc) of women?
- What were the impacts on the health of children? Was there increased incidence of malnutrition, illness, fatalities?

6. Over your lifetime, do you believe there has been a change in climate?

- Are long dry periods occurring more/less often? Are they occurring for longer?
- Are floods happening more or less often?
- Are days of extreme heat – that makes it difficult to be outside and continue regular activities – occurring more or less often?

7. What are some solutions and strategies the community has developed to reduce these impacts during long dry periods or floods?)

- What is done to reduce risk of disease and illness among women and children? among herders? and among elders?
- What is done to ensure sufficient food availability for all, particularly for children?
- What is done to manage insufficient water availability during such times?
- Are there changes to animal herd management including size, grazing patterns, water access during these periods?
- Do you think these strategies that have been employed to reduce risk are still effective? What would make them more effective?
- Are there any Government programs that help the community to become more resilient? How can these be improved?
- Are there any non-state actors (NGOs) programs that help the community to be more resilient?
- What resources, investments, knowledge would best build the reliance of the local people?

8. Over your lifetime, has there been more long-term change in your livelihood and cultural practices because of the changes in climate extremes?

# Appendix 4: Survey questions

*Because of the length of our survey, we include just questions highlighted in the main text.*

**“Go” participant questions**

- During the drought, what was the furthest fora [pasture with water] you went to?
  - Is that fora farther away than you usually go during the dry season? [44 of 45 “go” participants said “yes”]
    - [If yes] How much farther, in days of walking? [All 45 “go” participants answered this question; the person who said “no” above still said 2 days farther here]
- During the drought, how many months at a time were you away from your family?
  - Is that longer than you are usually away from your family? [All 45 “go” participants said “yes”]

**“Go” and “stay” participant questions**

- During the drought, were there long periods when you went hungry? [All 90 participants said “yes”
  - What did you do to deal with this? [This was an open-ended short response where participants could list multiple things. Responses were thematically coded as follows: sold animals (n = 88 reported this), slaughtered animals (n = 28), went into debt with a shop (n = 19), shared with a neighbor or friend (n = 16), engaged in casual labor (n = 6), did self-employed work (e.g., making beads or charcoal; n = 3), or relied on the government or an NGO (n = 4)]
- During the drought, did you experience any health impacts from the heat like headaches, extreme tiredness, skin problems, breathing problems, issues moving or thinking, anxiety, skin problems, or issues breathing?
  - 1, Headaches | 2, Extreme tiredness | 3, Issues breathing | 4, Dizziness | 5, Breathing problems | 6, Confusion | 7, Poor coordination | 8, Restlessness | 9, Anxiety | 10, Dry skin | 11, Swollen tongue | 12, sweating profusely | 88, Other specify [Note that confusion, poor coordination, restlessness, and swollen tongue were not reported by participants. We examine reports of stress and hopelessness from a different question focused on mental health impacts rather than reports of anxiety here]
- During the drought, did you feel/experience stress? [All 90 participants said yes]
- During the drought, did you feel hopelessness/helplessness - feeling "where you are going to start from"? [The latter phrase is how Rendille expressed feelings of hopelessness to us during pilot interviews. All 90 participants said “yes”]
